# Zika Virus Potential Vectors among *Aedes* Mosquitoes from Hokkaido, Northern Japan: Implications for Potential Emergence of Zika Disease

**DOI:** 10.3390/pathogens10080938

**Published:** 2021-07-24

**Authors:** Leo Uchida, Miki Shibuya, Ronald Enrique Morales-Vargas, Katsuro Hagiwara, Yasukazu Muramatsu

**Affiliations:** 1 School of Veterinary Medicine, Rakuno Gakuen University, Ebetsu, Hokkaido 069-8501, Japan; s21361159@g.rakuno.ac.jp (M.S.); k-hagi@rakuno.ac.jp (K.H.); y-mrmt@rakuno.ac.jp (Y.M.); 2 Department of Medical Entomology, Faculty of Tropical Medicine, Mahidol University, Ratchathewi, Bangkok 10400, Thailand; ronald.mor@mahidol.ac.th

**Keywords:** Zika virus, susceptibility, *Aedes japonicus*, *Aedes galloisi*, *Aedes* mosquitoes, Palearctic ecozone

## Abstract

The Zika virus (ZIKV) is a rapidly expanding mosquito-borne virus that causes febrile illness in humans. *Aedes aegypti* and *Ae. albopictus* are the primary ZIKV vectors; however, the potential vector competence of other *Aedes* mosquitoes distributed in northern Japan (Palearctic ecozone) are not yet known. In this study, the susceptibility to Zika virus infection of three *Aedes* mosquitoes distributed in the main city of the northern Japan and their capacities as vectors for ZIKV were evaluated. Field-collected mosquitoes were fed ad libitum an infectious blood meal containing the ZIKV PRVABC59. The Zika virus was detected in the abdomen of *Ae. galloisi* and *Ae. japonicus* at 2–10 days post infection (PI), and from the thorax and head of *Ae. galloisi* at 10 days PI, resulting in 17.6% and 5.9% infection rates, respectively. The Zika virus was not detected from *Ae. punctor* at any time. Some northern Japanese *Aedes* could be suspected as vectors of ZIKV but the risk may be low when compared with major ZIKV vectors.

## 1. Introduction

There are more than 3500 species of mosquito classified in 112 genera [1]; mosquitoes are one of the most important vectors of arboviruses such as Dengue virus (DENV), Zika virus (ZIKV) and Chikungunya virus (CHIKV). In Japan, approximately 112 species of mosquitoes are distributed from north to south; they are divided into two groups: 40 species of northern mosquitoes and 72 species of southern mosquitoes, which are distributed in Palearctic ecozone and Indomalaya ecozone, respectively [2]. In the field of arbovirus research, most infectious experiments that use live mosquitoes have been based on the use of two of southern mosquitoes, *Aedes aegypti* and *Aedes albopictus*, because of their importance as major vectors of several arboviruses. In addition, laboratory colonies of both species have been well established and the easy handling of these laboratory specimens has contributed to useful arbovirus research. Other mosquito species, especially northern mosquitoes whose colonies are not well established, have rarely been used for research because of the difficulty of the experiments. Thus, little is known about the arbovirus vector competence (VC) of northern *Aedes* mosquitoes and their importance in the transmission cycle.

The ZIKV, a member of family *Flaviviridae*, is a mosquito-borne virus that causes acute febrile illness in humans. Since the Zika outbreak in South America from 2015 to 2016, the relationship between ZIKV infection and microcephaly in neonatal humans has been confirmed and this has become a concern for public health [3,4,5,6,7]. During an outbreak, ZIKV is maintained in a human and mosquito cycle; humans get infected primarily from mosquito bites, although other transmission routes such as sexual and transfusion-mediated transmission have been reported [8]. *Ae. aegypti* and *Ae. albopictus* are considered major vectors of ZIKV and other arboviruses such as DENV and CHIKV [9]. In addition to these two major vectors, epidemiological studies clarified that *Aedes hensilli* and *Aedes polynesiensis* could have been vectors of ZIKV during the outbreaks in the Yap Islands and French Polynesia, respectively [10,11]. Moreover naturally ZIKV-infected *Aedes* species, such as *Ae. africanus*, *Ae. dalzieli*, *Ae. furcifer*, *Ae. luteocephalus*, *Ae. vittatus* have been reported [12,13,14]. Experimental ZIKV infection studies have been conducted by several researchers using *Aedes* mosquitoes to evaluate their VC [15,16,17]. According to the review written by Epelboin Y. et al., total 13 *Aedes* mosquitoes have been evaluated their VC for ZIKV, and 7 species, *Ae. aegypti*, *Ae. albopictus*, *Aedes camptorhynchus*, *Aedes luteocephalus*, *Aedes notoscriptus*, *Aedes vexans* and *Aedes vittatus* are competent to ZIKV [12]. However, little is known about the VC of Japanese *Aedes* mosquitoes especially which distribute in Palearctic ecozone.

In 2014, DENV was introduced into Japan from abroad after 70 years with no confirmed domestic cases. In Tokyo, the DENV outbreak, which was spread by local *Ae. albopictus*, resulted in 160 autochthonous cases [18,19]. This outbreak proved that a foreign emerging or reemerging arbovirus can easily cross borders and cause outbreaks that are maintained by local mosquito vectors. To date, three imported cases of ZIKV have been reported in Japan, although there are no confirmed domestic cases [20]. In this study, three species of field-collected northern Japanese mosquitoes, from the Palearctic ecozone, were challenged with ZIKV strain PRVABC59, and the virus titer in the mosquito body parts was determined to evaluate ZIKV susceptibility. In addition, we found a unique amino acid substitution in domain III of the viral envelope, which was observed in a newly propagated virus within a mosquito abdomen.

## 2. Results

### 2.1. Species of Collected Mosquitoes and Flavivirus Screening

**Figure 1:**
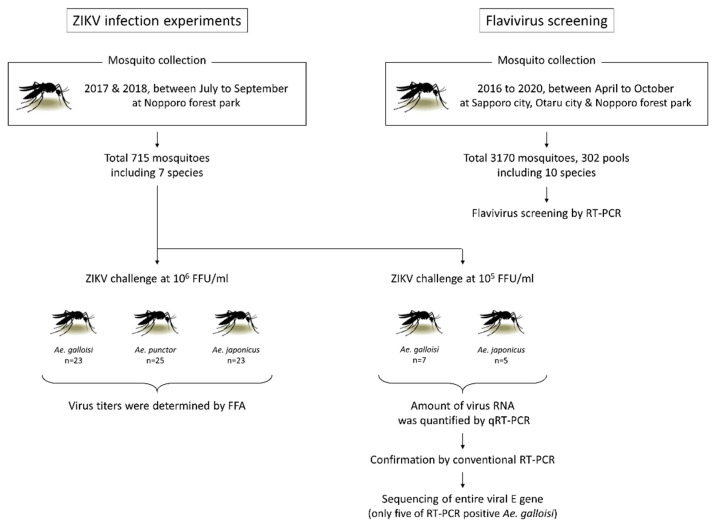
Flow chart of the experimental procedure. For the ZIKV infection experiments, 715 mosquitoes were collected in Nopporo forest park between July and September in both 2017 and 2018. All 715 mosquitoes were orally challenged with an IBM containing 10^5^ FFU/mL or 10^6^ FFU/mL of the ZIKV strain PRVABC59, and 158 mosquitoes ingested an IBM. A part of IBM-ingested *Ae. punctor*, *Ae. japonicus* and *Ae. vexans* were used for another preliminary experiments. The virus titers and the amount of virus RNA were determined by FFA and qRT-PCR, respectively. Conventional RT-PCR was used to confirm the qRT-PCR results, and the sequence of entire viral E gene of RT-PCR positive samples was determined. In another study 3170 mosquitoes were collected between April and October in 2016 to 2020 to screen the naturally infected Flaviviruses. Three hundred two pools including 10 species were screened by RT-PCR and the sequence of amplicons were determined.

Flow chart of the experiments is shown in Figure 1. Between July and September in both 2017 and 2018, 715 mosquitoes were collected in Nopporo forest park, Hokkaido Prefecture, Japan (Figure S1). Of the 715 mosquitoes, 311 were *Ae. japonicus*, 127 were *Ae. punctor*, 99 were *Ae. galloisi*, 27 were *Ae. vexans*, seven were *Tripteroides bambusa*, five were *Ae. bekkui*, and four were *Culex orientalis*. According to Kamimura’s categorization, all seven species belong to northern mosquitoes that distribute in the Palearctic ecozone in Japan [2]. All 715 mosquitoes were orally challenged with an infectious blood meal (IBM) containing 10^5^ FFU/mL or 10^6^ FFU/mL of the ZIKV strain PRVABC59, and 158 mosquitoes ingested an IBM (Table 1). Another 135 mosquitoes could not be identified morphologically but were not confirmed by species using cytochrome c oxidase unit I (*COI*) gene sequencing because they did not ingest the IBM and were excluded from the further experiments.

To exclude the possibility of natural infection of ZIKV in collected mosquitoes, pan-Flavivirus screening was conducted by reverse transcription PCR (RT-PCR), although the domestic ZIKV case has not been reported in Japan. Between April and October in 2016 to 2020, 3170 mosquitoes (302 pools) were collected in Sapporo city, Otaru city and Nopporo Forest Park, consisting of seven species of *Aedes*, *An. sineroides, Cx. orientalis* and *Cx. pipiens* group (Table 2). In all pools, ZIKV RNA was not detected. In the two pools of *Cx. pipiens* group, Culex flavivirus (CxFV) which distribute in *Culex* spp. worldwide was detected, but not in *Aedes* and *Anopheles* pools (Table 2). These results indicated that the possibilities of natural ZIKV infection in northern mosquitoes and viral focus formation by another Flaviviruses are extremely low or negligible.

### 2.2. Ae. galloisi and Ae. japonicus Demonstrate Susceptibility to ZIKV Strain PRVABC59

**Figure 2:**
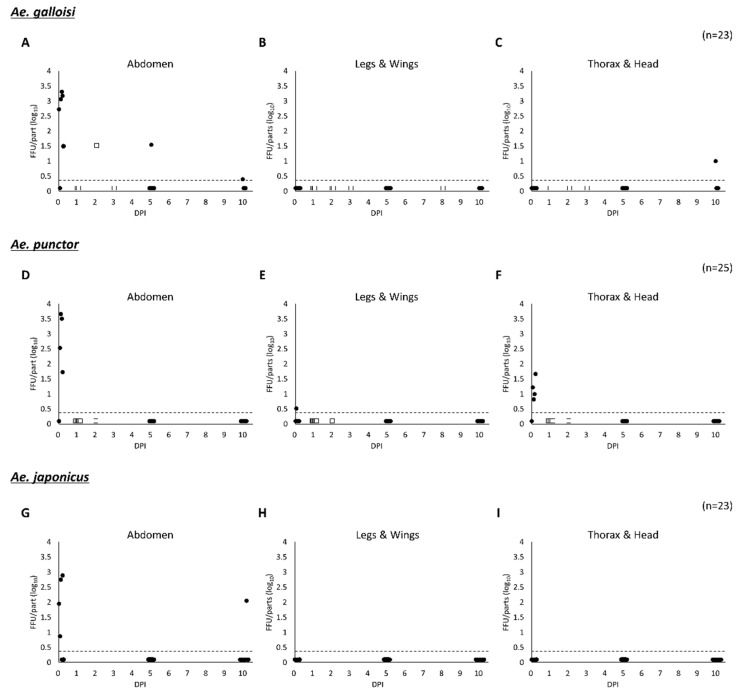
ZIKV titer in the virus-challenged three species of mosquitoes. Three species of mosquitoes, *Ae. galloisi* (**A**–**C**), *Ae. punctor* (**D**–**F**), *Ae. japonicus* (**G**–**I**) were fed an IBM containing 10^6^ FFU/mL of ZIKV strain PRVABC59. The virus titers in three body parts; abdomen, thorax and head, and the legs and wings of both naturally dead (□) and killed (●) mosquitoes were determined by FFA. The broken lines in the figure indicate the detection limit of this assay. Markers occurring below the broken lines indicate that any focus was not observed from the part (i.e., below the detection limit). Detailed individual titer data are available in Table S2. DPI: days post infection.

First, ZIKV susceptibility was evaluated in three species of *Aedes* mosquitoes. The data of *Ae. bekkui* was excluded from the further study because of the limitation of the mosquito number. The mosquitoes were challenged with 10^6^ FFU/mL of ZIKV strain PRVABC59 and killed at 5- and 10-days post infection (PI) by freezing at −80 °C. Mosquitoes that died naturally were also used for the experiment. To confirm the virus inoculation and evaluate the amount of inoculated virus, some mosquitoes were killed after blood feeding immediately (0 days PI). In all three species, the average amount of inoculated virus was 2.56 ± 0.86 (log10) FFU/abdomen and there were no significant differences among the species (Figure 2 and Table S2). ZIKV was not detected from any body parts of *Ae. punctor* after 1-day PI even though they took ZIKV into their abdomen via ingestion of an IBM. In *Ae. galloisi*, ZIKV was detected from the abdomen at 2, 5 and 10 days PI, and from thorax and head at 10 days PI. It should be noted that in the same *Ae. galloisi*, ZIKV was detected from abdomen, thorax and head at 10 days PI (Table S2). In *Ae. japonicus*, ZIKV was detected from the abdomen at 10 days PI. The infection rates (excluding 0 days PI) were 17.6% and 5.9% in *Ae. galloisi* and *Ae. japonicus*, respectively, and there was no statistically significant difference in infection rates between two species. On the other hand actual infection rate of *Ae. punctor* was unrevealed, and the possibility that *Ae. punctor* shows low infection rate of less than 5% cannot be rule out due to the limitation of each sample size. In summary, the two mosquito species showed some susceptibility to the ZIKV strain PRVABC59.

### 2.3. Ae. galloisi Shows Higher Susceptibility to a Smaller amount of ZIKV Challenge When Compared with Ae. japonicus

**Figure 3:**
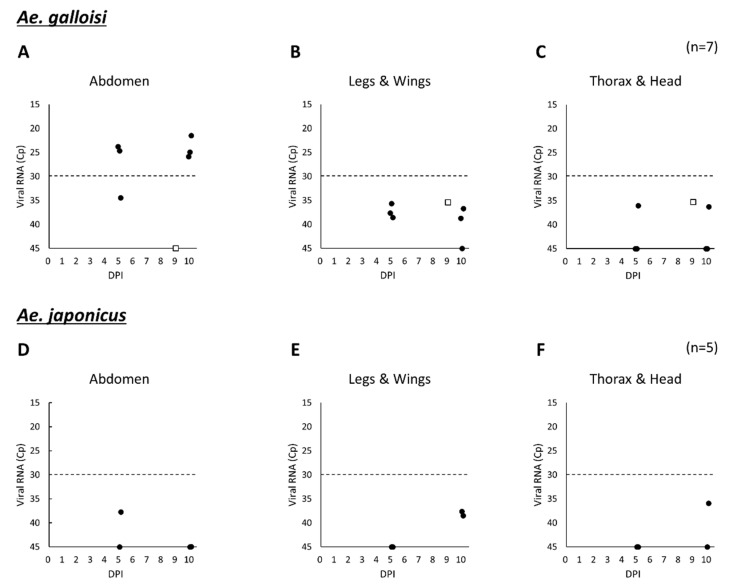
Amount of ZIKV RNA in the virus-challenged *Ae. galloisi* and *Ae. japonicus*. *Ae. galloisi* (**A**–**C**) and *Ae. japonicus* (**D**–**F**) were fed an IBM containing 10^5^ FFU/mL of ZIKV strain PRVABC59. The amounts of virus RNA in three body parts; abdomen, thorax and head, and the legs and wings of both naturally dead (□) and killed (●) mosquitoes were quantified by qRT-PCR. The broken lines in the figure show the cut-off value of Cp. The detailed individual Cp value is available in Table S3. DPI: days post infection.

When considering the above results, a lower amount (10^5^ FFU/mL) of ZIKV was given to *Ae. galloisi* and *Ae. japonicus* to compare their susceptibility to ZIKV. In both species, viral RNA was detected by quantitative RT-PCR (qRT-PCR) (Figure 3) at 5 and 10 days PI. To confirm the results, we conducted conventional RT-PCR targeting the viral envelope (E), but contrary to our expectations, only five samples, which showed low Cp values using qRT-PCR, were positive (Table S3). Additionally the specificity of the qRT-PCR was confirmed by using uninfected *Ae. galloisi* and *Ae. japonicus*, but even in these mosquitoes, some amplifications were observed with high Cp value. According to these results and the modest Cp value, 30 was set as the cut-off value of qRT-PCR (Figure 3). High Cp values observed in *Ae. japonicus* and some *Ae. galloisi* were considered as a results of unspecific reaction.

In *Ae. japonicus*, ZIKV infection was not confirmed, however, 71.4% of *Ae. galloisi* had ZIKV RNA in their abdomens at 5 and 10 days PI (Figure 3). It is suggested that *Ae. galloisi* is more susceptible to ZIKV strain PRVABC59 than *Ae. japonicus* although the number of samples is limited.

### 2.4. ZIKV Could Propagate in the Abdomen of Ae. galloisi

**Figure 4:**
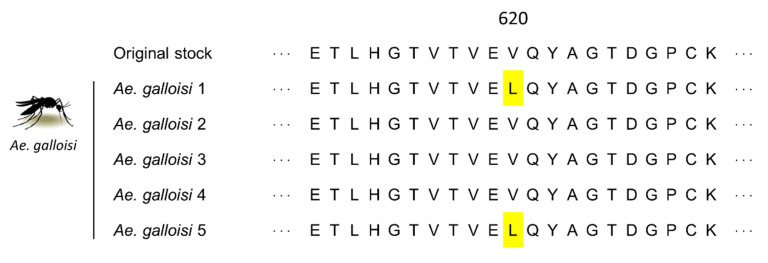
Multiple alignment of the partial envelope amino acid sequences. Amino acid sequences of the envelope (E) region derived from the original stock of ZIKV strain PRVABC59 and the viruses detected from abdomen of *Ae. galloisi* (*Ae. galloisi* 1 to 5) were aligned using the multiple alignment tool, CLC Sequence Viewer v7.6. In the entire E region of *Ae. galloisi*-derived viruses, a single nucleotide substitution (G1937T) causing amino acid substitution (V620L) was observed. The nucleotide and amino acid positions are based on ZIKV strain PRVABC59 (GenBank accession no. KX087101.3).

To clarify whether the detected ZIKV in mosquitoes was a residue of the inoculated virus or newly propagated virus, sequencing of the entire viral E gene was conducted. If the sequences completely match the original virus stock, it is suggested that the inoculated virus remained in the mosquito abdomen. In the entire E region of *Ae. galloisi*-derived viruses, two samples showed a single nucleotide substitution (G1937T) when compared with the original stock. Moreover, the nucleotide substitution causes amino acid change from valine (V) to leucine (L) at position 620 (Figure 4). The substitution is not likely to be caused by chance or error of the RT-PCR because the same nucleotide substitutions were observed at the same position among different mosquitoes, suggesting that the ZIKV was newly propagated in the mosquito body but not a residue of the inoculated virus. The sequence chromatograms are shown in Figure S3.

## 3. Discussion

In this study, the infectious ZIKV strain PRVABC59 was detected from the abdomens of *Ae. galloisi* and *Ae. japonicus* between 2 and 10 days after viral challenge. Moreover, the virus was detected from thorax and head of *Ae. galloisi* at 10 days PI. However, the infectious virus was not isolated from *Ae. punctor*. VC is defined as “the capacity of a mosquito to acquire the pathogen and support its transmission” [21]. Our results suggested that both *Ae. galloisi* and *Ae. japonicus* are susceptible to ZIKV, and ZIKV could be replicated in *Ae. galloisi*. However, the actual transmission ability could not be proved in this study due to the lack of an evidence of virus existence in their saliva. Further investigation evaluating virus titration in saliva and salivary gland provide insight into their actual VC for ZIKV in both species.

To date, among the three mosquito species, ZIKV susceptibility or VC has been evaluated in only *Ae. japonicus* [15]. According to Jansen S. et al. [15], *Ae. japonicus* captured in Germany showed higher infection rates, 66.7%, when compared with our results of 5.9%. Additionally, their virus titer in mosquitoes reached 5.9 ± 1.8 (log10) RNA copies/organ, while our mosquitoes showed 2.05 (log10) FFU/body part [15]. On the other hand Abbo S. R. et al. have reported that the virus was detected from 10% of *Ae. japonicus* body after 14 days post oral challenge of IBM containing 1.6 × 10^7^ 50% tissue culture infective dose per milliliter of ZIKV strain Suriname 2016 [22]. One of the reasons for this difference might be the inoculated virus amounts. In the study described here, 10^6^ FFU/mL of ZIKV was spiked in the IBM, while higher titer of ZIKV was spiked in the IBM in their study [15,22]. Other factors include differences in mosquito strain and virus strain. Even within the same mosquito species, great variability in VC has been reported in *Ae. aegypti* and *Ae. albopictus* against ZIKV infection [23,24,25]. Recently, *Ae. japonicus* has invaded from Japan and Korea to North America, Hawaii, Europe and other Asian countries [26]. It is possible that the native *Ae. japonicus* and invasive *Ae. japonicus* have different susceptibilities against ZIKV, like the cases of *Ae. aegypti* and *Ae. albopictus* [27].

When considering the registered genome sequences of ZIKV strain PRVABC59 in GenBank, three of the twelve genomes have valine and the remaining nine genomes have leucine at position 620 in the envelope. This suggests that the position itself is unstable and the virus seems to exist as a quasi-species [28]. In an additional experiment, the ZIKV strain PRVABC59 was sequentially passaged by monkey-derived Vero and mosquito-derived C6/36 to evaluate the stability of the position. The experiment was performed in triplicate, and after five passages, one of the C6/36-passaged samples showed the same amino acid substitution, V620L at the position. However, the remaining two C6/36-passaged samples and all Vero-passaged samples did not (Figure S3). Interestingly, the sequence chromatogram clarified the population shift from valine type to leucine type in five passages by C6/36 (Figure S3). The amino acid at position 620 exists in the beta-strand at domain III of Flavivirus envelope [29,30], and the domain seems important for the binding between the virus and cellular receptors [31,32]. To date there are no reports suggesting relationships between V620L substitution and any viral infectivity, but it is possible that the substitution is correlated with host cell preference and adaptability.

Several important ZIKV vectors such as *Ae. albopictus*, *Ae. aegypti*, *Ae. hensilli* and *Ae. polynesiensis* belong to subgenus *Stegomyia* [33,34]. Especially *Ae. albopictus* and *Ae. aegypti* are considered the most important vectors in human endemic infections [35]. ZIKV has been isolated from non-Stegomyia mosquitoes such as *Aedes opok* [36], *Aedes vittatus* and *Aedes furcifer* [13] in addition to non-*Aedes* mosquitoes such as *Anopheles coustani* and *Culex perfuscus* [13], although their VC is unclear. In this study, both *Stegomyia* (*Ae. galloisi*) and non-*Stegomyia* (*Ae. punctor* and *Ae. japonicus*) mosquitoes were used for the experiments and it should be noted that the *Ae. punctor* did not show any susceptibility to ZIKV (Figure 2). *Ae. galloisi* showed a higher susceptibility to the lower ZIKV challenge when compared with *Ae. japonicus* (Figure 3). At 0-day PI, i.e., immediately after virus intake, infectious ZIKV was detected from the abdomen of and *Ae. punctor*. The ZIKV did not maintain infectivity in the abdomen for 5 days and the virus was eliminated from *Ae. punctor*. VC in non-*Stegomyia Aedes* has not been well characterized, but Hart C. E. et al. reported that *Aedes* (*Ochlerotatus*) *taeniorhynchus* did not have susceptibility to ZIKV strain MEX 1–44 at the same dose of virus challenge in this study [16]. Moreover, Hall-Mendelin et al. reported that non-*Stegomyia Aedes*, such as *Aedes notoscriptus*, *Aedes procax* and *Aedes vigilax*, did not show VC although ZIKV was identified in the body after 14 days post virus challenge [17]. On the other hand, another study showed evidence that ZIKV was shed in saliva of *Ae. notoscriptus* after 14 days post virus challenge, and there is no consensus about the VC in this species [37]. Further studies focusing on the evaluation of ZIKV VC in several non-*Stegomyia Aedes* species may provide insight into the mechanisms of VC differences.

Among approximately 130 *Aedes* mosquito species, little is known about the VC for ZIKV, and most species have not been evaluated yet. In this study, the ZIKV susceptibility of two northern *Aedes* mosquitoes, *Ae. galloisi* and *Ae. japonicus*, was confirmed. However, their actual VC, i.e., the ability of virus transmission to a new host, is still unclear. Further experiments focused on the VC and the evaluation of transovarial transmission, and the establishment of the laboratory colonies will help improve the understanding of the risk of northern *Aedes* mosquitoes as a vector of invasive ZIKV.

## 4. Materials and Methods

### 4.1. Cells and Viruses

Rhesus monkey (*Macaca mulatta*)-derived LLC-MK2, African green monkey (*Cercopithecus aethiops*)-derived Vero and mosquito (*Aedes albopictus*)-derived C6/36 cells were maintained in a minimum essential medium (MEM) supplemented with 10% fetal bovine serum (FBS) and an antibiotic cocktail consisting of penicillin, streptomycin and amphotericin B. The LLC-MK2 and Vero cells were maintained at 37 °C with 5% CO_2_ and the C6/36 cells were maintained at 28 °C without CO_2_. ZIKV strain PRVABC59 (GenBank accession no. KU501215) was obtained from Dr. S. Tajima (National Institute of Infectious Diseases, Tokyo, Japan). The strain was isolated from a human who traveled to Puerto Rico in 2015 [38]. For making the virus stock, the ZIKV was inoculated to Vero cells at multiplicity of infection (MOI) of 0.01 and propagated for 3 to 4 days. The propagated virus titer was determined by focus forming assay (FFA) using LLC-MK2 cells according to previously described methodologies [39]. The virus stocks were kept at −80 °C for further experiments.

### 4.2. Mosquito Collection, Maintenance and Species Identification

For the virus infection experiment, mosquitoes were collected between July and September in 2017 and 2018 at the Nopporo Forest Park (43°04′19.0″ N 141°30′40.8″ E) in Ebetsu City, Hokkaido Prefecture, Japan using the human landing catch method and insect nets. The mosquito collection was conducted from 13:00 to 20:00 (almost from 18:00 to 19:30) on a sunny day. The mosquitoes landed on the clothes or captured by nets were transferred into a mosquito rearing device (one mosquito/device) and maintained in a 12 h:12 h light: dark cycle at 26 ± 2 °C and 65 to 85% relative humidity (Figure S2), and used for the virus infection experiment within 24 h after collection. In another study, for the screening of naturally-infected Flaviviruses, mosquitoes were collected between April and October in 2016 to 2020 at Sapporo city (43°03′04.9″ N 141°18′58.6″ E), Otaru city (43°14′13.7″ N 141°00′43.1″ E) and Nopporo Forest Park using the above method. The map of the sample collection sites and the climatic data are shown in Figure S1.

All of the mosquitoes were identified based on morphological characteristics [40]. In some mosquitoes that were difficult to identify by morphological characteristics, DNA barcoding based on *COI* gene was conducted according to methods described in a previous study [41].

### 4.3. Experimental ZIKV Infection of Mosquitoes

ZIKV was spiked into the horse erythrocytes to inoculate the mosquitoes. Anticoagulated horse blood (Nippon Bio-Test Laboratories Inc., Asaka, Saitama, Japan) was washed with phosphate buffered saline (PBS) twice and a stock solution of ZIKV was mixed with erythrocytes, after adjusting the volumes with MEM supplemented with 10% FBS (called infectious blood meal, IBM). Two hundred microliters of the IBM were transferred into the handmade reservoir and sealed using pork sausage casings (Matsunaga Incs., Toda, Saitama, Japan) (Figure S2). The reservoir was placed on a heat block and heated at 39 to 40 °C. The rearing devices, each containing a mosquito, were placed on the reservoir for 5 min in the dark to enable them to feed on the IBM. Stereomicroscope was used to judge whether the mosquito in the rearing device fed on IBM or not. Partially blood-fed, i.e., not fully engorged mosquitoes were also counted as IBM-feeding. The feeding rate was calculated by dividing the “number of IBM-feeding” by the “number of collection”. The IBM-fed mosquitoes were fed with 2% sucrose and 2% honey solution ad libitum and maintained for up to 10 days PI in the above described conditions. Mosquitoes that died naturally and mosquitoes that were killed were kept at −80 °C for further species identification, virus isolation and RNA extraction.

### 4.4. Virus Titration and Viral RNA Evaluation in the Mosquitoes

Three body parts of the mosquitoes; abdomen, thorax and head, and the legs and wings were separated using a microscope and were then homogenized in 200 µL of MEM supplemented with 2% FBS using a pestle. The thorax and head, and the legs and wings were not separated but mixed as one part, respectively. The homogenate was passed through 0.22 µm centrifuge tube filters (Corning, Corning, NY, USA) to remove the residue, and the filtrate was titrated by FFA as described above.

In another study, viral RNA was evaluated by RT-PCR and qRT-PCR. Total RNA was extracted from the three body parts or pooled mosquitoes (up to 20 mosquitoes) using RNeasy Mini kit (Qiagen, Hilden, DE-NW, Germany). Each sample was homogenized in 350 µL of buffer RLT with a stainless bead using a Retcsh MM300 TissueLyser (Qiagen) at the speed of 30 Hz for 4 min. The homogenate was passed through the RNeasy Mini Spin Column and RNA was purified according to the manufacturer’s protocol. First-strand cDNA was synthesized by SuperScript III Reverse Transcriptase (Thermo Fisher Scientific, Waltham, MA, USA). Thirteen microliters of the reaction mixture consisted of 1 µL of 10 µM ZIKV or Flavivirus specific reverse primer (Table S1), 1 µL of 10 mM dNTP Mix and 11 µL of extracted RNA. The mixture was incubated at 65 °C for 5 min and incubated on ice for 1 min. Four microliters of First-Strand buffer, 1 µL of 0.1 M dithiothreitol, 1 µL of RNase OUT and 1 µL of SuperScript III RT were added to the mixture, and the mixture was incubated at 50 °C for 20 min, 52.5 °C for 20 min, 55 °C for 20 min and 70 °C for 15 min. PCR was performed in 25 µL of the total reaction mixture containing 2.5 µL of Ex Taq buffer, 2 µL of 10 mM dNTP Mix, 1.25 µL of 10 µM forward and reverse primer (Table S1), 0.125 µL of Ex Taq (Takara Bio Inc., Otsu, Osaka, Japan) and 3 µL of first-stranded cDNA. The PCR thermal cycle condition for ZIVK detection was a single cycle at 94 °C for 2 min, followed by 30 cycles at 98 °C for 10 s, 55 °C for 30 s, 72 °C for 2 min, and a final extension at 72° for 10 min. On the other hand the thermal cycle condition of Flavivirus screening was referred by previous study [42]. The PCR products were electrophoresed on a 1.5% agarose gel at 100 V for 30 to 40 min to examine the product sizes. Sequencing of the PCR products was performed by the FASMAC sequencing service (Fasmac Co., Ltd., Atsugi, Kanagawa, Japan).

The qRT-PCR was performed using Thunderbird Probe One-step qRT-PCR Kit (Toyobo, Osaka, Japan) in accordance with previously described techniques [43]. Twenty-five microliters of reaction mixture consisted of 12.5 µL of reaction buffer, 0.625 µL of DNA Polymerase, 0.625 µL of RT Enzyme Mix, 1.25 µL of 10 µM reverse and forward primer (Table S1), 0.5 µL of 10 µM probe and 3 µL of RNA. The qRT-PCR was performed with the Light Cycler 480 System II (Roche, Basel, Switzerland) according to the following thermal cycle conditions; a single cycle at 50 °C for 10 min and 95 °C for 1 min, followed by 55 cycles at 95 °C for 15 s, 60 °C for 45 s, and cooling at 40 °C for 10 s. At the end of the extension step, fluorescence intensity (Em/Ex = 618/660 nm) was measured and crossing point (Cp) value was calculated by Light Cycler 480 Software v1.5.1.

### 4.5. Data Analysis

To evaluate species differences, mosquito infectivity was compared using the Fisher’s exact test and average value of the virus titer was compared using the *t*-test with the statistical analysis software EZR v2.14 (Easy R) [44].

## Figures and Tables

**Table 1:** Species and number of collected mosquitoes and engorged mosquitoes.

Species	No. of Collection	No. of IBM-Feeding	Feeding Rate
*Ae. bekkui*	5	5	100.0%
*Ae. galloisi*	99	30	30.3%
*Ae. punctor*	127	29	22.8%
*Ae. japonicus*	311	79	25.4%
*Ae. vexans*	27	15	55.6%
*Cx. orientalis*	4	0	0.0%
*Tr. bambusa*	7	0	0.0%
Unidentified	135	0	0.0%
Total	715	158	22.1%

**Table 2:** Screening of naturally infected Flaviviruses in northern mosquitoes.

Species	No. of Collection	No. of Pool	Detected Flavivirus (No. of Positive Pools)
*Ae. ezoensis*	183	21	0
*Ae. galloisi*	1	1	0
*Ae. japonicus*	2619	198	0
*Ae. nipponicus*	18	5	0
*Ae. punctor*	1	1	0
*Ae. togoi*	134	22	0
*Ae. vexans*	74	10	0
*An. sineroides*	1	1	0
*Cx. orientalis*	37	14	0
*Cx. pipiens* group	86	19	CxFV (2) *
Unidentified	16	10	0
Total	3170	302	2

* CxFV: Culex flavivirus.

## Data Availability

All data are available in the manuscript and Supplementary Materials.

## Supplementary Materials

The following are available online at https://www.mdpi.com/article/10.3390/pathogens10080938/s1, Figure S1: Map of Hokkaido Prefecture showing the sample collection site and the climatic data, Figure S2: Mosquito rearing device and IBM reservoir, Figure S3: Sequence chromatogram of Vero-passaged and C6/36-passaged ZIKV at the amino acid position 620, Table S1: Sequence of primers for RT-PCR and qRT-PCR, Table S2: ZIKV titer in mosquito body parts, Table S3: ZIKV RNA load in mosquito body parts.
