# Supplementary material for: Zika Virus Potential Vectors among Aedes Mosquitoes from Hokkaido, Northern Japan: Implications for Potential Emergence of Zika Disease
Source: Pathogens. 2021 Jul 24;10(8):938. doi: 10.3390/pathogens10080938 (PMC8399329; doi:10.3390/pathogens10080938)
Supplement: Supplementary file 1 [file pathogens-10-00938-s001.zip › Figure S1.pdf]

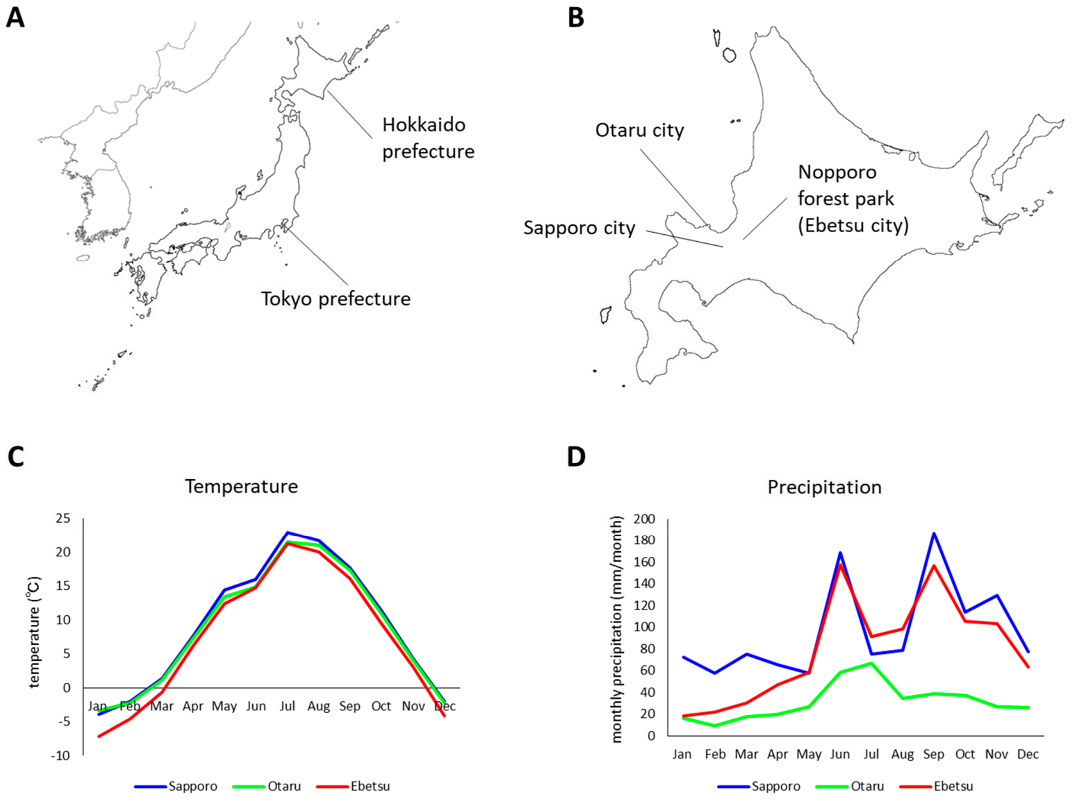

Figure S1. Map of Hokkaido Prefecture showing the sample collection site and the climatic data.

The map shows the site of Hokkaido Prefecture in Japan (A) and the mosquito collection site, Sapporo city (43°03'04.9"N 141°18'58.6"E), Otaru city (43°14'13.7"N 141°00'43.1"E) and Nopporo Forest Park (43°04'19.0"N 141°30'40.8"E) in Ebetsu City, Hokkaido Prefecture (B). The line graph shows the monthly average temperature (C) and the total monthly precipitation (D) in the above three sites in 2017. The climatic data was obtained from the web site of Japan Meteorological Agency (https://www.jma.go.jp/jma/indexe.html).
