# Supplementary material for: Zika Virus Potential Vectors among Aedes Mosquitoes from Hokkaido, Northern Japan: Implications for Potential Emergence of Zika Disease
Source: Pathogens. 2021 Jul 24;10(8):938. doi: 10.3390/pathogens10080938 (PMC8399329; doi:10.3390/pathogens10080938)
Supplement: Supplementary file 1 [file pathogens-10-00938-s001.zip › Figure S2.pdf]

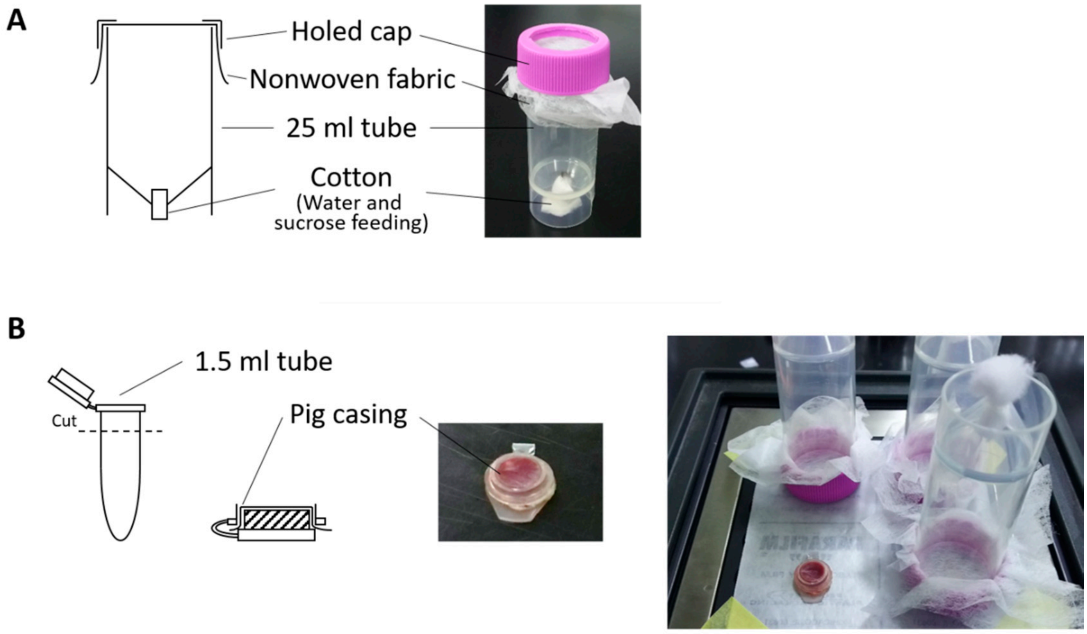

Figure S2. Mosquito rearing device and IBM reservoir.

(A) A hole was bored into the cap and the bottom of 25 ml conical tube using a utility knife and drill, respectively. A double-layered, nonwoven fabric was interposed between the cap and tube. (B) A 1.5 ml tube was cut at the position and 200 μl of IBM was applied into the cap side. The reservoir was covered by pork sausage casings and heated at 39 to 40°C on the heat block. The mosquito rearing device was put on the reservoir to allow the mosquitoes to feed on blood from the reservoir.
