# Supplementary material for: Zika Virus Potential Vectors among Aedes Mosquitoes from Hokkaido, Northern Japan: Implications for Potential Emergence of Zika Disease
Source: Pathogens. 2021 Jul 24;10(8):938. doi: 10.3390/pathogens10080938 (PMC8399329; doi:10.3390/pathogens10080938)
Supplement: Supplementary file 1 [file pathogens-10-00938-s001.zip › Figure S3.pdf]

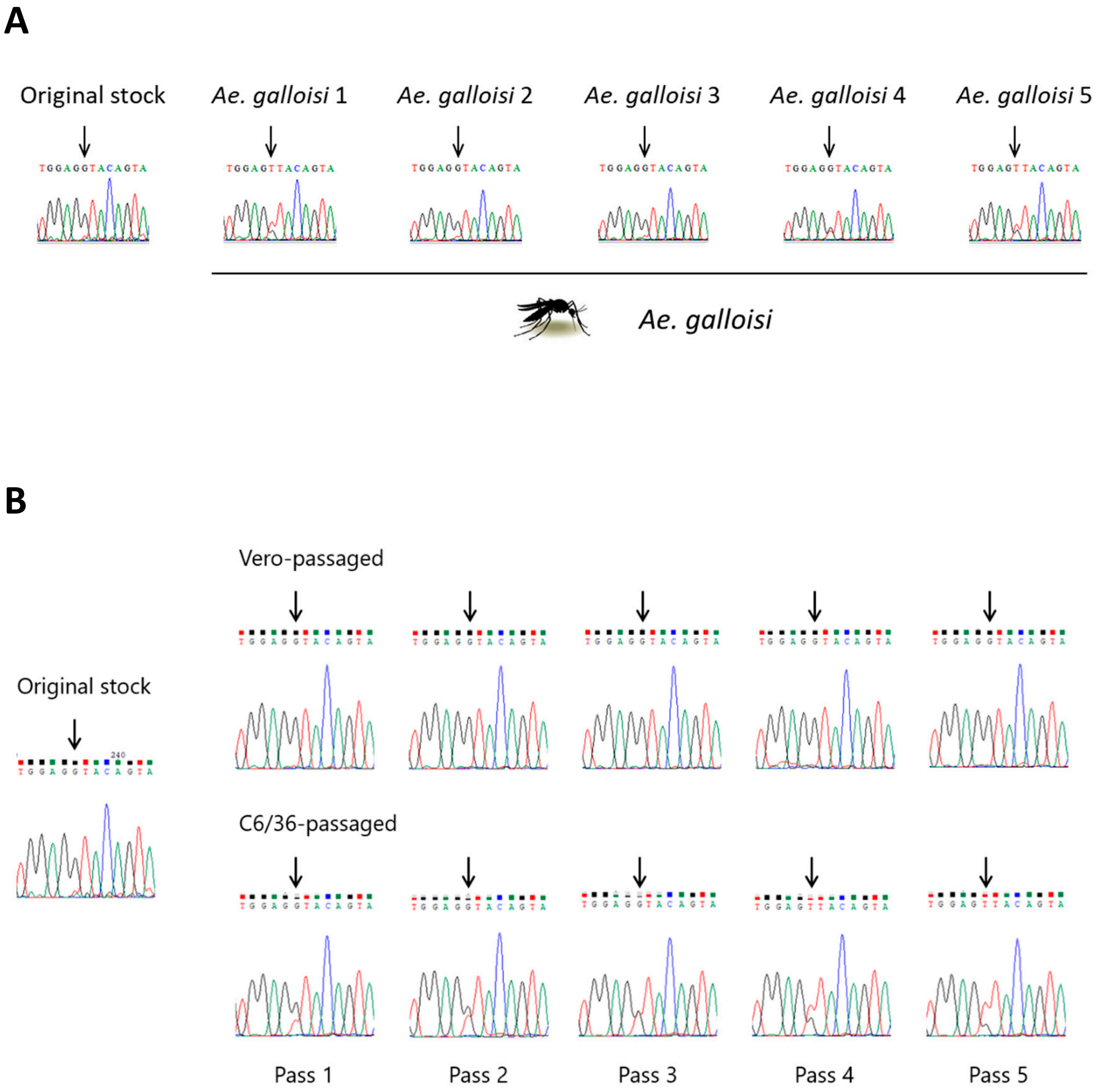


**Figure S3.** Nucleotide sequence chromatogram of ZIKV at the amino acid position 620. (A) Entire E region derived from the original stock of ZIKV strain PRVABC59 and the viruses detected from abdomen of *Ae. galloisi* (*Ae. galloisi* 1 to 5) were sequenced. (B) ZIKV strain PRVABC59 was sequentially passaged five times using Vero and C6/36 cells. After each passage, the total RNA was extracted from infectious culture fluid and the sequence of the entire viral envelope was determined. Black arrows show the position of single nucleotide substitution (G1937T) causing amino acid substitution (V620L). The nucleotide and amino acid positions are based on ZIKV strain PRVABC59 (GenBank accession no. KX087101.3).
