# Supplementary material for: Zika Virus Potential Vectors among Aedes Mosquitoes from Hokkaido, Northern Japan: Implications for Potential Emergence of Zika Disease
Source: Pathogens. 2021 Jul 24;10(8):938. doi: 10.3390/pathogens10080938 (PMC8399329; doi:10.3390/pathogens10080938)
Supplement: Supplementary file 1 [file pathogens-10-00938-s001.zip › Table S1.pdf]

Table S1. Sequence of primers for RT-PCR and qRT-PCR.

Purpose	Primer or probe	Sequence (5'-3')[a]	Location	Source
Pan-Flavivirus RT-PCR	MAMD	aacatgatgggRaaRagRgaRaa	NS5	[42]
	cFD2 mix	gtgtcccaBccDgcKgtRtcatcIgc	NS5	
ZIKV E RT-PCR	MR766 902-921	tggcttttgggaagctcRac	prM	This study[b]
	MR766 2576-2557	ccctccaggcttcaacRtcR	NS1	
ZIKV quantification (qRT-PCR)	ZIKV 1086	ccgctgcccaacacaag	E	[43]
	ZIKV 1162c	ccactaacgttcttttgcagacat		
	ZIKV 1107-Cy5	Cy5-agcctaccttgacaagcagtcagacactcaa-BHQ3		

[a] R, B, D, K indicate mixed nucleotides and I indicate inosine. [b] Nucleotide positions are based on Zika virus strain MR766 (GenBank accession no. KY989511.1).
