# Supplementary material for: Zika Virus Potential Vectors among Aedes Mosquitoes from Hokkaido, Northern Japan: Implications for Potential Emergence of Zika Disease
Source: Pathogens. 2021 Jul 24;10(8):938. doi: 10.3390/pathogens10080938 (PMC8399329; doi:10.3390/pathogens10080938)
Supplement: Supplementary file 1 [file pathogens-10-00938-s001.zip › Table S2.pdf]

Table S2. ZIKV titer in mosquito body parts.

Species	Date of natural dead (PI)	Date of killed (PI)	Viral titer[a] FFU/body parts (log_{10})		
			Abdomen	Legs & Wings	Thorax & Head
Ae.galloisi		0	2.73		
(n=23)		0			
		0	3.06		
		0	3.32		
		0	3.18		
		0	1.50		
	1				
	1				
	1				
	2		1.52		
	2				
	3				
		5	1.54		
		5			
		5			
		5			
		5			
		5			
	8				
		10	0.40		1.00
		10			
		10			
		10			
Ae. punctor		0	2.54	0.52	1.22
(n=25)		0	3.67		0.82
		0	3.51		1.00
		0	1.73		1.67
		0			
	1				
	1				
	1				
	1				
	1				

	1		
	2		
		5	
		5	
		5	
		5	
		5	
		5	
		10	
		10	
		10	
		10	
		10	
		10	
		10	
Ae. japonicus		0	1.95
(n=23)		0	0.88
		0	2.76
		0	2.89
		0	
		0	
		5	
		5	
		5	
		5	
		5	
		5	
		5	
		10	2.05
		10	
		10	
		10	
		10	
		10	
		10	
		10	
		10	
		10	

[a] Blanks in the columns of viral titer indicate that no infectious virus was identified from the body parts by FFA. PI: post infection.
