# Supplementary material for: Zika Virus Potential Vectors among Aedes Mosquitoes from Hokkaido, Northern Japan: Implications for Potential Emergence of Zika Disease
Source: Pathogens. 2021 Jul 24;10(8):938. doi: 10.3390/pathogens10080938 (PMC8399329; doi:10.3390/pathogens10080938)
Supplement: Supplementary file 1 [file pathogens-10-00938-s001.zip › Table S3.pdf]

Table S3. ZIKV RNA load in mosquito body parts.

Species	Date of natural dead (PI)	Date of killed (PI)	Viral RNA Cp value of qRT-PCR[a]		
			Abdomen	Legs & Wings	Thorax & Head
Ae. galloisi		5	34.50	38.56	36.05
(n=7)		5	24.67	35.66	
		5	23.78	37.64	
	9			35.43	35.30
		10	21.47	36.69	36.28
		10	25.88	38.73	
		10	24.91		
Ae. japonicus		5	37.71		
(n=4)		5			
		10		38.52	35.95
		10		37.63	

[a] Blanks in the columns of viral RNA indicate higher Cp value over 45. PI: post infection.
